# Coix Seed Diet Ameliorates Immune Function Disorders in Experimental Colitis Mice

**DOI:** 10.3390/nu14010123

**Published:** 2021-12-28

**Authors:** Qilyu Zhou, Ruyang Yu, Tianlong Liu, Yeye Li, Jia Zhong, Tao Zhang, Zhongjie Liu, Yusheng Hu

**Affiliations:** 1 College of Veterinary Medicine, China Agricultural University, Beijing 100193, China; zhou7l@163.com (Q.Z.); ptyhqfish@163.com (R.Y.); liutianlong@cau.edu.cn (T.L.); bluelihuohuo@163.com (Y.L.); liuzhongjiecau@163.com (Z.L.); 2 College of Veterinary Medicine, Shanxi Agricultural University, Jinzhong 030801, China; zhongjia3294@163.com; 3 Beijing Key Laboratory of Traditional Chinese Veterinary Medicine, Beijing University of Agriculture, Beijing 102206, China; zhangtao@bua.edu.cn

**Keywords:** Coix seed, T-lymphocyte subsets, innate immune cells, dextran sulfate sodium, ulcerative colitis

## Abstract

Coix seed is a functional food in the Chinese diet that possesses the ability to alleviate ulcerative colitis clinically. However, the underlying mechanisms remain ambiguous. In this study, we investigated the protective effect of the Coix seed diet on experimental colitis mice. The mice were randomly divided into four groups: control group, model group, Coix seed feed group, and positive control group. The maintenance feed of the mice was replaced with Coix seed feed 10 days before orally administering the mice 5% (*w*/*v*) dextran sulfate sodium drink. As a result, the Coix seed feed alleviated colitis symptoms, maintained the complete blood count at a normal level, reduced the pathological score, relieved inflammatory cytokine secretion, and alleviated oxidative stress. Network pharmacology analysis was used for further exploration of the targets of Coix seed feed. The results showed that T-cell regulation is one of the targets of Coix seed feed, and the analysis of the T-lymphocyte subset and innate immune cell distribution of the colon tissue supported the network pharmacology results. In conclusion, Coix seed, as a staple food, can alleviate experimental colitis, and the mechanism may be related to the immune regulation effect of Coix seeds.

## 1. Introduction

Ulcerative colitis (UC) is a gut disease that is characterised by abdominal pain, diarrhoea, or constipation. Patients with UC often exhibit poor quality of life and psychological health because of the chronic and recurrent features of the disease. The pathogenesis of UC is multifactorial and includes genetic predisposition, immune system dysfunction, psychological factors, and environmental factors. Studies have shown that the coordination between tumour necrosis factor-α (TNF-α) and interleukin- 6 (IL-6) is a crucial factor for UC onset and development [1], and interleukin- 10 (IL-10) exerts an inflammation inhibition effect during UC progression [2]. The three cytokines are regulated by nuclear factor kappa B (NF-κB), which is a multiple function inflammatory factor [1]. Inflammatory reactions may generate reactive oxygen species (ROS), which may in turn activate NF-κB and induce severe damage [3]. Clinically, oxidative stress in the gastrointestinal system of patients with UC increases, indicating that the oxidative stress is another vital index of UC [4]. Thus, exploring an approach to regulate the inflammatory reactions in the colon to alleviate UC is essential.

Induction of the inflammatory reaction is a mechanism of foreign matter clearance by the immune system. An appropriate inflammatory reaction could help maintain homeostasis; however, excessive or deficient inflammatory reactions can be tissue-damaging or even life-threatening. Mucosal immune dysfunction plays a pivotal role in the occurrence and maintenance of UC [5,6], and an appropriate immune modulator may help alleviate UC symptoms.

Current medical management is to prevent patients from colectomy and colorectal cancer (CRC). Clinical drugs such as 5-aminosalicylic acid (5-ASA), corticosteroids, thiopurines, biological drugs, and anti-TNF-α drugs are most commonly used to treat UC; however, the side effects of these drugs should not be ignored [6].

Coix seeds are commonly consumed as staple grain in China because of their mild pharmacology effects. In traditional Chinese medicine theory, Coix seeds are commonly used for inflammation treatment, and they also produce antipholgistic and antitumor effects [7]. Studies have reported that Coix seeds could alleviate colitis clinically and could prevent liver cancer by regulating the immune system [8,9]; however, whether Coix seeds could regulate the immune system during UC remains ambiguous. In Chinese medicine research, network pharmacology is an emerging analytical method for predicting the gene targets and pathways involved in the interaction between drugs and diseases. In the present study, we investigated the anti-colitis effect of Coix seeds, explored the mechanism through the network pharmacology analysis, and conducted experiments to identify the potential biological process.

## 2. Materials and Methods

### 2.1. Preparation of Coix Seed Feed (CSF)

Coix seeds were ground into ultrafine powder (φ ≤ 200 μm) and then sent to Xiao Shu You Tai Biotechnology Co., Ltd. (Beijing, China) for Coix seed feed (CSF) production. In the CSF, Coix seed ultrafine powder completely replaced corn bran. The detailed nutritional ingredients of the CSF and maintenance feed are shown in Table 1.

### 2.2. Animal Experiments

Eight-week-old female Kunming mice (weight, 37 ± 2 g), purchased from Sipeifu Co. Ltd. (Beijing, China), were used as the experiment animals. The mice were raised under 22 ± 2 °C temperature, 50% ± 10% relative humidity, and a 12 h light–dark cycle. The animal experiments were conducted after approval from the China Agricultural University Laboratory Animal Care and Use Ethics Committee (AW15012020-3).

The mice were randomly divided into four groups, namely control group (CON), model group (dextran sulfate sodium, DSS), CSF group, and positive control group (5-ASA), with each group containing eight mice. The mice in the CON, DSS, and 5-ASA groups were fed with the maintenance feed purchased from Xiao Shu You Tai Biotechnology Co., Ltd. (Beijing, China), whereas the CSF group mice were fed with the CSF for 10 days before modelling. The 5-ASA group was fed with Mesalazine SR Granules (Ipsen, Boulogne-Billancourt, France) (200 mg/kg × d) for 10 days before modelling. Afterwards, all the groups, except the control group, were provided with 5% (*w*/*v*) dextran sulfate sodium (DSS) drink every two days, the CSF and 5-ASA are continuously given until the end of the experiment. Specifically, the DSS drink was fed on days 11, 12, 15, 16, 19, and 20, and pure water was fed on days 13, 14, 17, and 18. The disease activity index (DAI) was evaluated on days 13, 17, and 21, according to the following features: occult blood (from 0 to 3, normal, hemoccult+, hemoccult++, bleeding); stool consistency (from 0 to 3, normal, with shape but cannot be picked, semifluid, watery diarrhoea). The occult blood assay was performed using a faecal occult blood test kit that was procured from BaSO Biotechnology Co. (Guangdong, China). At the end of the experiment, the mice were administered 2% pentobarbital sodium and then sacrificed. Blood samples of each mice was collected and sent to China Agricultural University Veterinary Teaching Hospital for complete blood counting with a multispecies hematology analyzer (Nihon Kohden, Tokyo, Japan). The colon of each mouse was excised and preserved for subsequent analysis.

### 2.3. Histological Analysis

The colon tissues were preserved in 4% paraformaldehyde at room temperature for 24 h. After embedding in paraffin, the tissues were sliced into 4 μm sections, mounted on glass slides, and subjected to hematoxylin–eosin (H&E) staining. Subsequently, the colon tissues were evaluated histologically. The histology score was determined by summing up the scores of each of the following four histological features: inflammation infiltration (0 to 4, none to severe); crypt damage (0 to 2, none to crypt structure lost); oedema severity (0 to 2, none to severe); goblet cell hypertrophy (0 to 2, none to 67–100%).

### 2.4. Enzyme Linked Immunosorbent Assay and Oxidative Damage Assay

The colon tissues of the mice were grinded in 0.9% saline, and the supernatant was used for cytokine, superoxide dismutase (SOD) and malondialdehyde (MDA) assays. The enzyme linked immunosorbent assay (ELISA) MAXTM Deluxe Set kits of TNF-α, IL-6, IL-10 were bought from BioLegend Inc. (San Diego, CA, USA). The SOD and MDA kits were bought from Beyotime (Shanghai, China), and all the indices were measured according to the instructions provided in the instruction brochure.

### 2.5. Ultra-Performance Liquid Chromatography–Mass Spectrometry (UPLC–MS/MS) Analysis of CSF

The Coix seed ultrafine powder (8 g) was dipped and decocted in 300 mL double distilled water for 1 h. Subsequently, the decoction was diluted with double distilled water to a final volume of 200 mL. The sample was analysed using the ultra-performance liquid chromatography-electrospray ionisation-mass spectrometry (UPLC-ESI-MS/MS) system (UPLC, Shim-pack UFLC SHIMADZU CBM30A system; MS, Applied Biosystems 6500 Q TRAP). Triple quadrupole (QQQ) and LIT scans were performed on a triple quadrupole-linear ion trap mass spectrometer (API 6500 Q TRAP UPLC/MS/MS System), with an ESI turbo ion-spray interface, operating in the positive and negative ion modes and controlled by Analyst 1.6.3 software (AB Sciex).

### 2.6. Exploration of the Mechanism of CSF against Dextran Sulfate Sodium (DSS)-Induced Colitis

#### 2.6.1. CSF Target Acquisition

CSF components detected through UPLC-ESI-MS/MS were further selected based on the criteria of oral bioavailability ≥ 30% and drug likeness ≥ 0.18 by using the Traditional Chinese Medicine Database and Analysis Platform (TCMSP, https://www.tcmsp-e.com/, accessed on 10 December 2020.). Then, we searched the acquired compounds on the TCMSP for target acquisition.

#### 2.6.2. DSS-Induced Colitis Target Acquisition

The GeneCards (https://www.genecards.org/, accessed on 10 December 2020.) was used for DSS-induced colitis target acquisition. The search query was ‘DSS-induced colitis’.

#### 2.6.3. Protein–Protein Interaction Network Construction

The protein–protein interaction (PPI) network was constructed using the STRING database (https://www.string-db.org/, accessed on 10 December 2020.), and the interactions with the probabilistic association confidence score of ≥0.4 were preserved.

#### 2.6.4. Gene Ontology, Kyoto Encyclopedia of Genes and Genomes Pathway Enrichment Analysis, and Biological Biochemical Image Database Analysis

The Database for Annotation, Visualization and Integrated Discovery (DAVID, https://david.ncifcrf.gov/, accessed on 10 December 2020.) was employed for the gene ontology (GO) analysis, Kyoto Encyclopedia of Genes and Genomes (KEGG) pathway enrichment analysis, and Biological Biochemical Image Database (BBID) analysis. The items with a *p* value of <0.05 were considered statistically significant

### 2.7. Flow Cytometry Analysis of Colonic Immune Cell Authentication

The colon tissue was trypsinised for 1 h after excising it from the mice. Then, the colon cells were filtered using a 70-μm cell stainer, and trypsinisation was stopped by using phosphate buffered saline (PBS) containing 5% fetal bovine serum. Moreover, the cells were dyed in an orderly manner with CD3 FITC, CD8a PE, CD4 allophycocyanin, and CD45 BV421 for T-cell subset identification and then with CD11b PE, Ly-6G PE-Cy7, F4-80 PerCP-Cy5.5, and CD11c FITC for innate immune cell detection. The antibodies were procured from BioLegend Inc. (San Diego, CA, USA). Then, the cells were detected using a flow cytometer BD LSRII, Inc. (New York City, NY, USA).

### 2.8. Statistics

Statistical analysis was performed using SPSS 20.0 (IBM SPSS, Chicago, IL, USA) with one-way ANOVA. The diagrams were drawn using GraphPad Prism 8 (GraphPad, San Diego, CA, USA).

## 3. Results

### 3.1. CSF Alleviated the DSS-Induced Colitis Mice’s Symptoms

**Figure 1:**
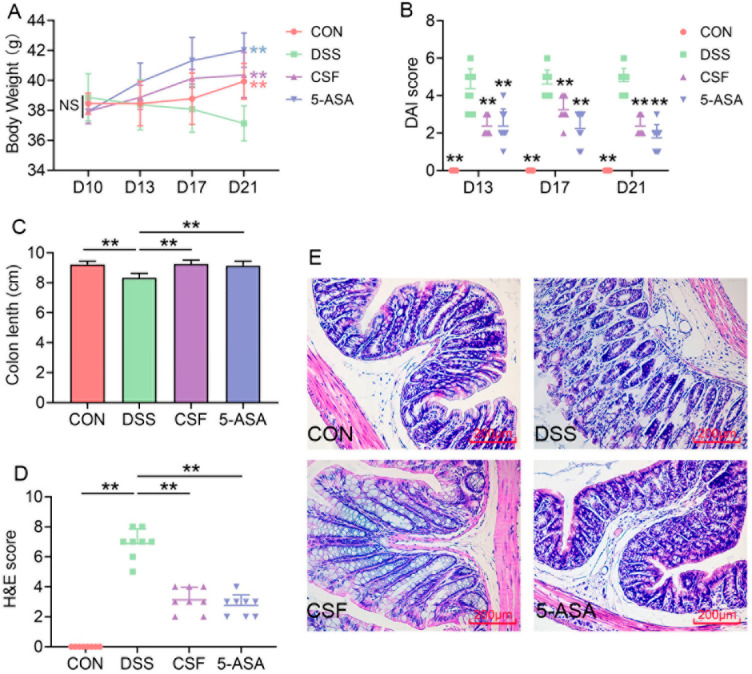
Coix seed feed (CSF) alleviated the symptoms of dextran sulfate sodium (DSS)-induced colitis mice (*n* = 8) (**A**) The bodyweight of mice in each group. (**B**) The disease activity index (DAI) score of each mouse. (**C**) The colon length of the mice in each group. (**D**) The hematoxylin–eosin (H&E) score of each mouse. (**E**) The H&E staining photo of the representative mouse in each group. The data are presented as mean ± SD, ** *p* < 0.01, the significance symbols in (**A**,**B**) show the significance compared with the DSS group.

In the present study, the body weight of the mice in the DSS group decreased, whereas that of the mice in the other groups increased significantly (Figure 1A). The DAI score indicated that the mice in the DSS group suffered from more severe colonic problems (diarrhoea, occult blood), whereas those in the CSF and 5-ASA groups exhibited significantly relieved symptoms (Figure 1B). Moreover, the colon length of the mice in the DSS group decreased significantly, whereas CSF and 5-ASA administration maintained the colon length in a normal range (Figure 1C). The H&E staining results showed that the microstructure was severely damaged in the DSS group and slightly damaged in the CSF and 5-ASA groups (Figure 1D,E).

### 3.2. CSF Ameliorated the Hemogram of the Colitis Mice

**Figure 2:**
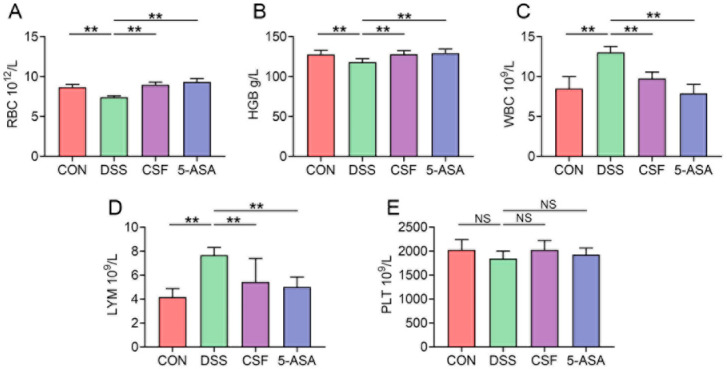
CSF changed the hemogram results of the colitis mice (*n* = 8). (**A**–**E**) The results of red blood cell (RBC) count, haemoglobin (HGB) level, white blood cell (WBC) count, lymphocyte (LYM) count, and platelet (PLT) count of the mice in each group. The data are presented as mean ± SD, ** *p* < 0.01, NS: no significance.

The red blood cell (RBC) count and haemoglobin (HGB) level of the DSS group decreased markedly, whereas those of the other two groups showed a remarkable increase compared with those of the CON group (Figure 2A,B). However, the white blood cell (WBC) and lymphocyte (LYM) counts demonstrated an opposite trend (Figure 2C,D), and no difference was observed in the platelet (PLT) count among the four groups (Figure 2E).

### 3.3. CSF Relieved the Inflammatory Cytokine Secretion and Oxidative Stress in the Colon Tissue of the Colitis Mice

**Figure 3:**
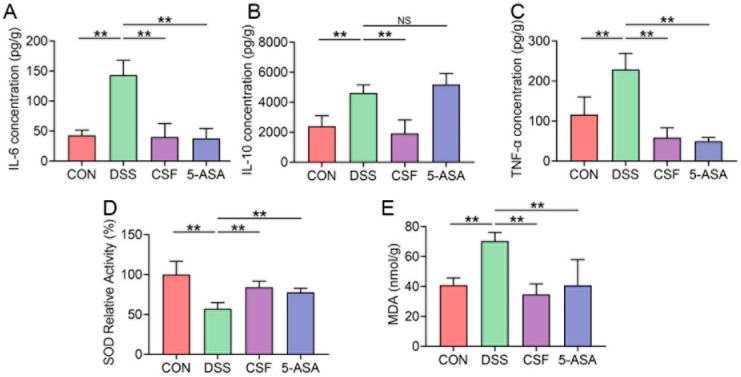
CSF relieved the colonic inflammatory cytokine secretion and oxidative stress in the colitis mice (*n* = 8). (**A**–**C**) The enzyme-linked immunosorbent assay (ELISA) results for interleukin- 6 (IL-6), IL-10, tumour necrosis factor-α (TNF-α) of the mice in each group. (**D**,**E**) The superoxide dismutase (SOD) and malondialdehyde (MDA) assays results of the mice in each group. The data are presented as mean ± SD, ** *p* < 0.01, NS: no significance.

The DSS group showed significantly higher IL-6 and TNF-α secretion than the other groups (Figure 3A,C). However, the anti-inflammatory cytokine IL-10 secretion in the DSS and 5-ASA groups was significantly higher than that in the other groups (Figure 3B). Furthermore, the DSS mice showed lower SOD activity but higher MDA activity than the other groups; the differences were significant (Figure 3D,E).

### 3.4. The Protein–Protein Interaction (PPI) Network Construction of CSF and DSS-Induced Colitis Mice

**Figure 4:**
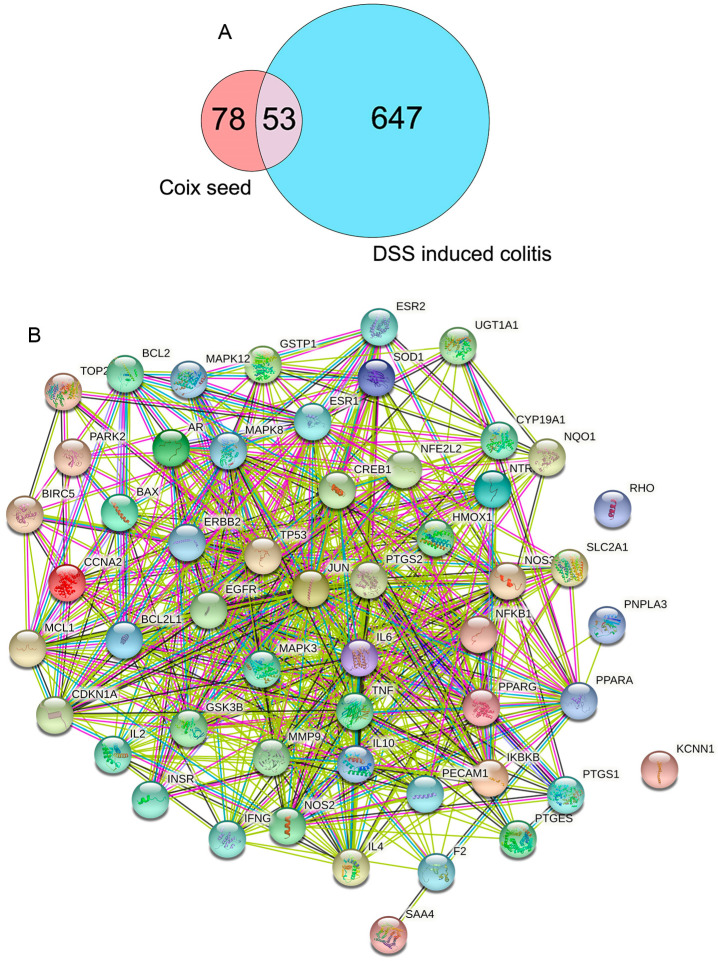
The gene targets of CSF and DSS-induced colitis. (**A**) The overlapping gene targets of CSF and DSS-induced colitis. (**B**) The protein–protein interaction (PPI) network of the 53 key targets constructed by STRING.

A total of 527 compounds were detected in the UPLC–MS/MS experiment, and 27 of these compounds met the set criteria (OB ≥ 30%, DL ≥ 0.18). These 27 compounds were further searched in the TCMSP, which resulted in the acquisition of 131 targets. On GeneCards, the query ‘DSS-induced colitis’ revealed 700 related targets. Overall, 53 overlapping targets were identified (Figure 4A), and the PPI network of the 53 targets was constructed with 53 nodes and 556 edges (Figure 4B).

### 3.5. The Gene Ontology (GO) and Kyoto Encyclopedia of Genes and Genomes (KEGG) Enrichment Analyses of 53 Targets in CSF against DSS-Induced Colitis

**Figure 5:**
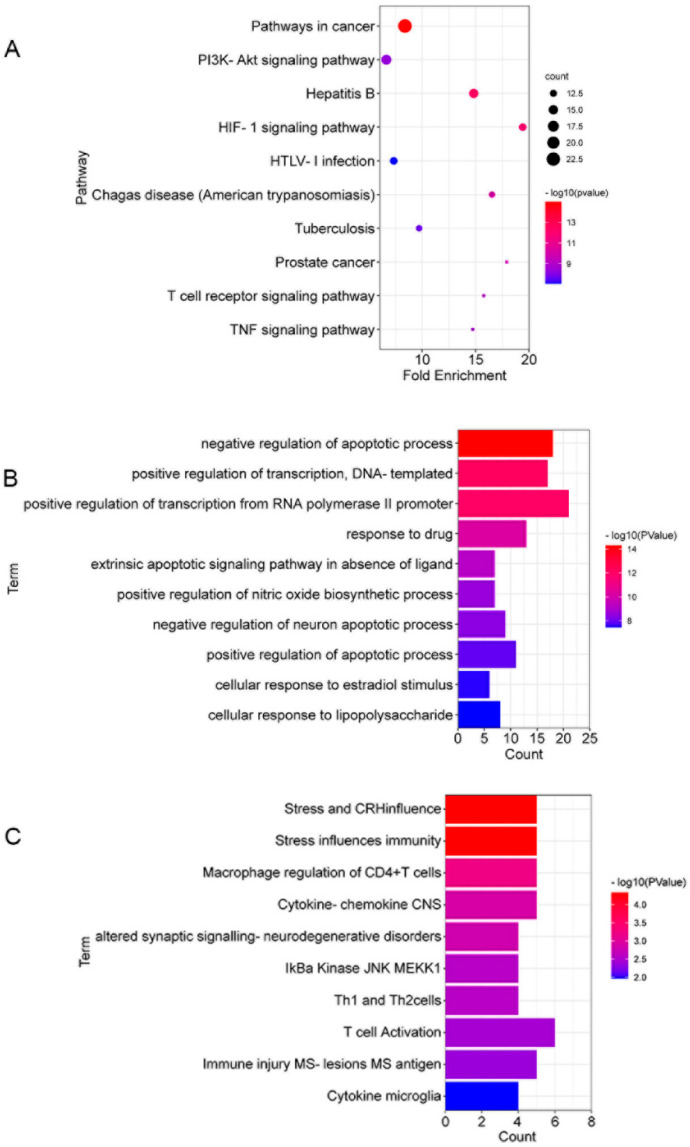
The gene enrichment analysis results of CSF against DSS-induced colitis. (**A**) The top 10 pathways (ranked by count) identified using the Kyoto Encyclopedia of Genes and Genomes (KEGG) pathway enrichment analysis. (**B**) The top 10 items (ranked by *p* value) identified using the gene ontology (GO) enrichment analysis. (**C**) The top 10 items (ranked by *p* value) identified using the BBID enrichment analysis.

To identify the underlying pathways involved in the protection mechanism of CSF against DSS-induced colitis, we used DAVID for pathway enrichment analysis. The results indicated the involvement of 103 KEGG pathways, 327 GO biological process items and 19 BBID items in the anti-colitis reaction of CSF. The KEGG pathway analysis indicated that the T-cell receptor signalling pathway and TNF signalling pathway were involved in the anti-colitis mechanisms of CSF. The GO enrichment analysis showed that the apoptotic process and cellular response to lipopolysaccharide (LPS) were involved in the CSF and DSS-induced colitis interaction. The BBID analysis showed that T-cell activation and regulation were involved in the interaction process. The top 10 KEGG pathways, GO items, and BBID items are presented in Figure 5A–C.

### 3.6. CSF Altered the T-Lymphocyte Subsets in the Colon Tissue of the Colitis Mice

**Figure 6:**
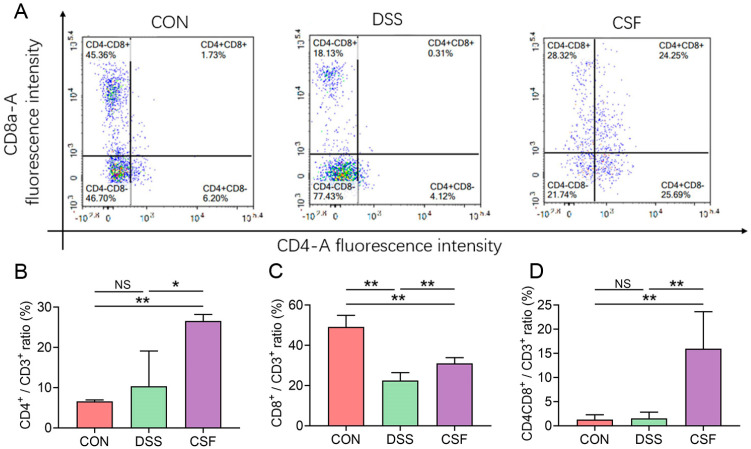
CSF altered the T-lymphocyte subsets in the colon tissue of the colitis mice (*n* = 5). (**A**) The flow cytometry results of T-lymphocyte subsets of the representative mouse. (**B**–**D**) The bar graph of the results. The data are presented as mean ± SD, * *p* < 0.05, ** *p* < 0.01, NS: no significance.

The CD4^+^/CD3^+^ ratio of the CSF group (26.56% ± 1.50%) was found to be markedly higher than that of the CON (6.62% ± 0.31%) and DSS (10.37% ± 7.83%) groups (Figure 6A,B), indicating that CSF upregulated the CD4^+^ expression in T lymphocytes residing in the colon tissue. The CD8^+^/CD3^+^ ratio of the CSF group (30.57% ± 2.33%) was significantly higher than that of the DSS group (22.43 ± 3.15%); however, both CSF and DSS groups displayed remarkably lower CD8^+^/CD3^+^ ratio than the CON group (49.27% ± 4.72%) (Figure 6A,C). CD4^+^CD8^+^ double-positive (DP) T lymphocyte is a newly discovered immune cell that expresses both CD4^+^ and CD8^+^ on the cell surface. The CD4^+^CD8^+^/CD3^+^ ratio of the CSF group (15.97% ± 6.85%) was significantly higher than that of the CON (1.28% ± 0.90%) and DSS (1.55% ± 1.15%) groups (Figure 6A,D).

### 3.7. CSF Changed the Innate Immune Cell Abundance in the Colon of the Colitis Mice

**Figure 7:**
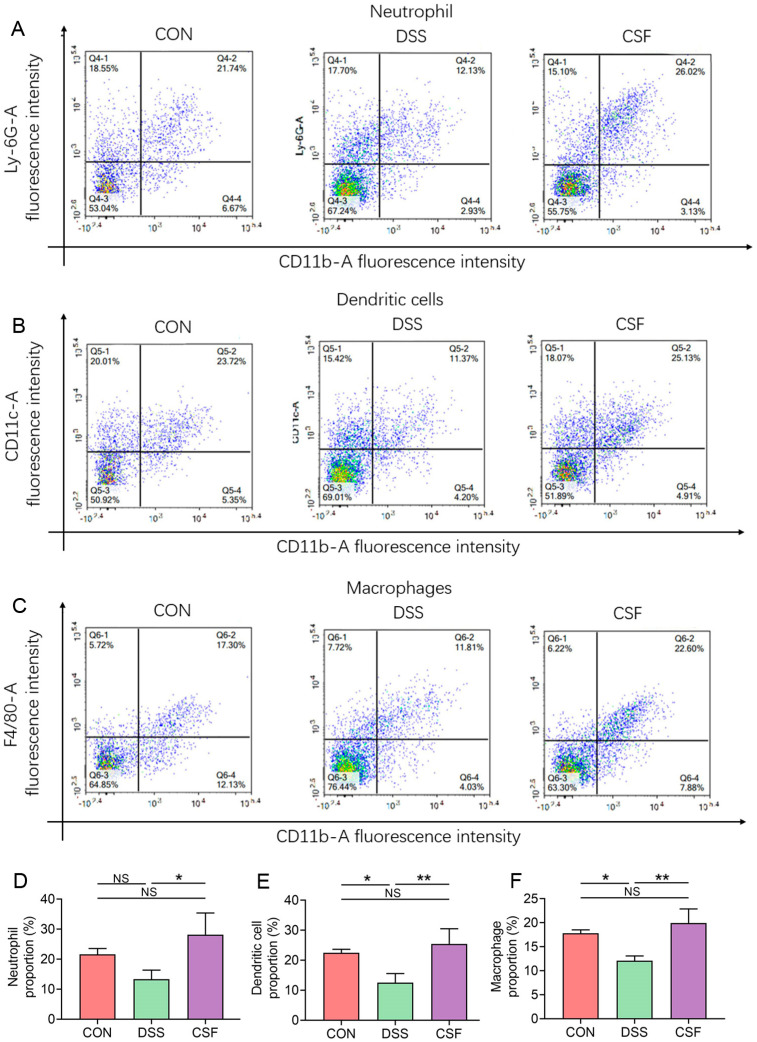
CSF changed the innate immune cell proportion in the colon of the colitis mice (*n* = 5). (**A**–**C**) Results for the abundance of neutrophils, dendritic cells, and macrophages in each group. (**D**–**F**) The bar graph of the three results. The data are presented as mean ± SD, * *p* < 0.05, ** *p* < 0.01.

CD11b-A is a commonly used surface marker for innate immune cells, and Ly-6G-A is a specific surface marker for mature neutrophils; therefore, the upper right quadrant of each group in Figure 7A represents the mature neutrophil proportion in innate immune cells in tissues. Likewise, CD11c-A is a specific marker for dendritic cells, and F4/80-A is a specific marker for macrophages; the upper right quadrant of Figure 7B,C represents the proportion of dendritic cells and macrophages, respectively.

The abundance of the colonic neutrophils in the CSF group (28.15% ± 6.45%) did not vary significantly with that in the CON group (21.59% ± 1.76%) but was significantly higher than that in the DSS group (13.30% ± 2.74%) (Figure 7A,D). The abundance of dendritic cells in the CSF group (25.46% ± 4.50%) did not differ with that in the CON group (22.50% ± 1.03%) but was remarkably higher than that in the DSS group (12.57% ± 2.66%) (Figure 7B,E). The abundance of colonic macrophages in the CSF group (19.95 ± 2.62%) showed no difference with that in the CON group (17.80% ± 0.63%); both groups exhibited remarkably higher abundance of colonic macrophages than the DSS group (12.10% ± 0.87%) (Figure 7C,F).

## 4. Discussion

UC is a severe gastrointestinal disease prevalent worldwide and is one of the greatest risk factors for CRC [5]. However, the available therapies provide unsatisfactory outcomes and are unable to meet physicians’ and patients’ expectations. Thus, in the present study, we determined the anti-colitis effect of Coix seed on mice. The mice in the CSF and 5-ASA groups exhibited increased body weight and a low DAI score and thus showed a better life quality. Moreover, the CSF maintained the colon length and pathological score at the same level as those in the 5-ASA group. Furthermore, the pathological observation indicated that the mice in the DSS group exhibited a disrupted micro structure, colonic fold atrophy, and oedema; the finding is consistent with those of a study [10], whereas CSF alleviated these pathological damages. The hemogram of the mice was tested to evaluate their physical condition. The DSS group mice suffered from anaemia, leukocytosis, and lymphocytosis and exhibited a decreasing tendency of PLT, consistent with the studies on clinical patients with UC [11]. However, the CSF could mitigate the aforementioned conditions, possibly by reducing the bleeding and inflammation in the colon. These results indicate that the CSF could alleviate DSS-induced colitis in mice. Therefore, we further explored the protection mechanisms of the CSF.

TNF-α is a core mediator of UC onset that activates the neutrophils and lymphocytes, and in turn, upregulates cytokine secretion in tissues. IL-6 is regarded as another major cytokine involved in UC progression because of its multiple functions in regulating the immunoreaction [12]. Moreover, studies have confirmed that TNF-α and IL-6 are overexpressed in UC patients. Therefore, we firstly determined the IL-6 and TNF-α concentrations in the colon tissue. The results showed that DSS caused TNF-α and IL-6 hypersecretion in the colon tissue, whereas CSF could inhibit the secretion of the two pro-inflammatory cytokines. SOD is a family of anti-oxidant enzymes, whose activity represents the anti-oxidation ability of the tissues. MDA is a lipid peroxidation product that induces oxidative stress in tissues. The two indices together reflect the oxidative stress in the colon tissue from both anti- and pro-inflammation aspects. Former studies have indicated that oxidative stress results from inflammatory reactions and is a major stimulation of UC [4]. Additionally, oxidative stress could activate NF-κB, a pro-inflammation factor, which in turn aggravate the inflammatory reaction [4]. In the present study, we estimated the oxidative stress level in the colon tissue of the mice. The results revealed that CSF and 5-ASA both alleviated oxidative stress in mice, which may be a CSF’s protective effect mechanism.

IL-10 is an anti-inflammation cytokine involved in colitis progression, and it plays an essential role in the recovery of tissues [13]. It is regulated by numerous factors, including lipid peroxidation products and pro-inflammatory cytokines. In our study, the IL-10 concentration of the DSS and 5-ASA groups increased significantly, which indicated the excessive inflammatory reaction and ROS activation. The IL-10 level was comparable between the CSF group and the CON group; however, the CSF group showed gentler symptoms clinically and pathologically compared with the DSS group, suggesting that the overexpressed IL-10 did not restrain the inflammatory damage in the colon. The aforementioned results confirmed that CSF exerted a protective effect on experimental colitis mice; however, the underlying mechanism remains unknown. Therefore, we further explored the anti-colitis mechanism of CSF through network pharmacology. The results indicated that the T-cell receptor signalling pathway is strongly associated with the CSF function against DSS-induced colitis; thus, we conducted further research on the residing T lymphocytes and the innate immune cells of the colon.

Inflammation is an efficient mechanism for the immune system to eliminate foreign matter and aetiology. A proper immune reaction could help maintain tissue homeostasis. Moreover, studies have demonstrated that the T lymphocytes undergo dysregulation during UC and experimental colitis [6]. Thus, we investigated how CSF regulates the T-lymphocyte subsets in the colon tissue during colitis in the present experiment. CD4 is a specific antigen that is mainly expressed by helper T lymphocytes. CD4^+^ T lymphocytes could regulate inflammatory reactions and protect the colon tissue during colitis [14]. Moreover, the mucosal barrier dysfunction is related to the lack of CD4^+^ T lymphocytes during the process of inflammation. In the present experiment, DSS attack showed a tendency of increase in the CD4^+^ T cell rate compared with that in the CON group, but CSF significantly alerted the index, indicating that CD4^+^ T cells may have an essential role in the protective effect mediated by CSF. In addition, CD4^+^ T cells can regulate immune cell differentiation, which may be the reason for a significant increase in CD8^+^ T cells in the CSF group compared with that in the DSS group. Although the CD8^+^ T cells differentiate from cytotoxic T lymphocytes, they are regarded as the tissue-protective cells that eliminate damaged or cancerous cells and clear space for tissue growth [15]. In our experiment, DSS attack dramatically decreased CD8^+^ T cells in colon mucosa, consistent with a clinical study [16]; however, the CSF upregulated CD8^+^ T cells in the experimental colitis mice, indicating a higher eliminating potential of the tissue. Recently, CD4^+^CD8^+^ DP T-lymphocytes have been found beneficial in the immune response of UC [17]. These lymphocytes are differentiated from CD4^+^ T lymphocytes and can maintain barrier integrity and eliminate damaged cells [18]. Moreover, the DP T-cell population was reported to decrease in CRC patients [18]. Our experiment showed that the CSF could increase the CD4^+^CD8^+^ DP T-cell rate in colitis mice, which may be due to the increased CD4^+^ T cells and contribute to the inflammatory regulation in DSS-attacked mice.

In addition to lymphocytes, innate immune cells play essential roles in inflammatory regulation. Although neutrophils are regarded as pro-inflammation cells, recent studies have unraveled their tissue-healing function [19,20]. Moreover, neutrophils could serve as an APC that activates the adaptive immune response [21]. Dendritic cells, as an interface between innate and adaptive immunity, are another potent APCs, whose reduction leads to T-cell immune suppression [22]. Recent studies have uncovered the dual functions of macrophages in host protection and pro-inflammation, which perfectly maintain the homeostasis of tissue [23]. In the mammalian intestine, macrophages constantly produce cytokines that regulate T-cell proliferation and differentiation [23]. Although the three cells are regulated by T-cell immunity, they also contribute to adaptive immune system activation, and their deficiency during inflammation results in more severe tissue pathology and even death [22,23,24]. In the present study, DSS decreased the rate of three cells, whereas the CSF maintained the indices at the same level as the CON group. Additionally, based on the network pharmacology results, we assume that CSF could regulate T-lymphocyte differentiation during DSS attack, thereby improving the innate immune cells recruitment and stimulating an accurate and efficient immune response to eliminate external biotics and damaged cells. Moreover, the anti-apoptosis effect and TNF signalling pathway regulatory effect may contribute to the protection of tissues. However, some pathways remain to be explored, and studies are warranted to investigate the mechanism for a persistently normal IL-10 expression level in the CSF group and whether there is correlation between the over expressed IL-10 and the decreased immune response.

## 5. Conclusions

The study demonstrated a protective effect of the CSF on DSS-induced colitis mice. Additionally, the experiments confirmed that the protective effect is associated with the immunity regulation effect of the CSF.

## Figures and Tables

**Table 1:** The detailed nutritional ingredients of the maintenance and Coix seed feed.

Ingredient	Maintenance Feed	Coix Seed Feed
crude protein (%)	20.00	18.29
crude fat (%)	4.50	4.06
crude fiber (%)	3.70	3.69
crude ash (%)	6.53	3.12
calcium (%)	1.19	1.28
phosphorus (%)	0.77	0.79
lysine (%)	0.93	0.86
methionine and cysteine (%)	0.67	0.56
arginine (%)	1.02	0.96
histidine (%)	0.50	0.48
tryptophan (%)	0.21	0.21
phenylalanine and phenylalanine (%)	0.51	1.44
threonine (%)	0.78	0.64
leucine (%)	1.59	1.43
isoleucine(%)	0.76	0.72
valine (%)	0.90	0.87
magnesium (%)	0.26	0.26
kalium (%)	0.64	0.64
Natrium (%)	0.32	0.32
vitamin K (mg/kg)	6.15	6.15
vitamin B1 (mg/kg)	16.00	16.00
vitamin B2 (mg/kg)	16.03	16.03
vitamin B6 (mg/kg)	10.43	10.43
nicotinic acid (mg/kg)	89.00	89.00
Pantothenic acid (mg/kg)	30.09	30.09
Folic acid (mg/kg)	7.50	7.50
Biotin (mg/kg)	0.28	0.28
vitamin B12 (mg/kg)	0.03	0.03
Choline (mg/kg)	1900.00	1900.00
iron (mg/kg)	180.00	180.00
manganese (mg/kg)	123.50	123.50
copper (mg/kg)	17.80	17.80
zinc (mg/kg)	56.20	56.20
iodine (mg/kg)	0.61	0.61
selenium (mg/kg)	0.16	0.16
vitamin A(KIU/kg)	10.70	10.70
vitamin D (KIU/kg)	1.50	1.50
vitamin E (KIU/kg)	103.00	103.00

## Data Availability

Not applicable.
